# Operationalising health systems thinking: a pathway to high effective coverage

**DOI:** 10.1186/s12961-020-00615-8

**Published:** 2020-11-03

**Authors:** Lara M. E. Vaz, Lynne Franco, Tanya Guenther, Kelsey Simmons, Samantha Herrera, Stephen N. Wall

**Affiliations:** 1 Population Reference Bureau, 1875 Connecticut Avenue, NW Suite 520, Washington, DC 20009 United States of America; 2 EnCompass LLC, 1451 Rockville Pike Suite 600, Rockville, MD 20852 USA; 3 Formerly with Save the Children US, 899 North Capitol St NE Suite 900, Washington DC, 20001 USA; 4 Ford Foundation, 320 E 43rd St, New York, NY 10017 USA; 5 Save the Children US, 899 North Capitol St NE Suite 900, Washington DC, 20001 USA

**Keywords:** Effective coverage, implementation strength, newborn health, health systems thinking

## Abstract

**Background:**

The global health community has recognised the importance of defining and measuring the effective coverage of health interventions and their implementation strength to monitor progress towards global mortality and morbidity targets. Existing health system models and frameworks guide thinking around these measurement areas; however, they fall short of adequately capturing the dynamic and multi-level relationships between different components of the health system. These relationships must be articulated for measurement and managed to effectively deliver health interventions of sufficient quality to achieve health impacts. Save the Children’s Saving Newborn Lives programme and EnCompass LLC, its evaluation partner, developed and applied the Pathway to High Effective Coverage as a health systems thinking framework (hereafter referred to as the Pathway) in its strategic planning, monitoring and evaluation.

**Methods:**

We used an iterative approach to develop, test and refine thinking around the Pathway. The initial framework was developed based on existing literature, then shared and vetted during consultations with global health thought leaders in maternal and newborn health.

**Results:**

The Pathway is a robust health systems thinking framework that unpacks system, policy and point of intervention delivery factors, thus encouraging specific actions to address gaps in implementation and facilitate the achievement of high effective coverage. The Pathway includes six main components – (1) national readiness; (2) system structures; (3) management capacity; (4) implementation strength; (5) effective coverage; and (6) impact. Each component is comprised of specific elements reflecting the range of facility-, community- and home-based interventions. We describe applications of the Pathway and results for in-country strategic planning, monitoring of progress and implementation strength, and evaluation.

**Conclusions:**

The Pathway provides a cohesive health systems thinking framework that facilitates assessment and coordinated action to achieve high coverage and impact. Experiences of its application show its utility in guiding strategic planning and in more comprehensive and effective monitoring and evaluation as well as its potential adaptability for use in other health areas and sectors.

## Contribution to the literature

- Health systems literature provides a wide array of conceptual models; however, a gap remains in linking these models to improvements at the point of intervention delivery – where effective coverage happens.
- The Pathway to High Effective Coverage fills this gap, unpacking the black box of ‘implementation strength’ while linking national policies and health systems structures, in operational and understandable ways, to household-, community- and facility-level interventions.
- We describe how the Pathway to High Effective Coverage evolved and its utility and adaptability for strategic planning, monitoring and evaluation.
- The Pathway to High Effective Coverage contributes to health systems strengthening literature by providing an approach to operationalise health systems thinking into strategic planning, monitoring and evaluation.

## Background

Reducing mortality and morbidity rates requires the implementation of evidence-based, high-impact interventions within health service delivery platforms that reach populations in need, with sufficient quality to achieve intended health outcomes. The global health community has, until recently, tracked measures of intervention coverage – specifically, the proportion of those in need who had contact with an intervention [1]. This simple measure of coverage does not capture the quality of the intervention provided. The recognition that previous measures of intervention coverage are inadequate to monitor progress towards reducing mortality and morbidity has driven a more nuanced concept. The term ‘effective coverage’ is now increasingly used in place of coverage, in recognition that health systems provide maximum benefit when those needing interventions can access high-quality services [2–6]. Discussions are occurring throughout the global health community on how to define and measure effective coverage for a wide range of health interventions. In the past 2 years alone, global working groups led by experts at WHO, the United Nations Fund for Children (UNICEF), Johns Hopkins University, the London School of Hygiene and Tropical Medicine, and others have all contributed work on this topic [4, 6, 7] (internal documentation).

The term ‘implementation strength’ has also entered the global health lexicon to describe the supply and demand-side conditions required to ensure effective coverage of an intervention [8–10]. Some have described implementation strength as the quantity of the intervention implemented [11–13], defined in terms of availability and use of staff, equipment and supplies at point of intervention, and supervision. Others have framed implementation strength as a measure of the intensity of implementation [14–17]. Measuring the implementation strength of interventions has been posited both as an approach to identify gaps in intervention delivery and as points for course correction [8]. Weak implementation of proven effective interventions can impede their impact, leading to what some have termed “*empty scale-up*” [18]. Therefore, effective coverage and implementation strength are key, interlinked factors required to achieve national level impact.

Achieving high effective coverage depends, in part, on the structures and functioning of health systems at all levels, including national, subnational, health facility and community, from establishment and rollout of policies to their effective translation into intervention delivery and coverage. These health systems exist within a broader context of social, political and economic systems that affect their functionality [19]. Many health systems models and frameworks describe and analyse health systems and delivery of maternal, newborn and child health interventions. WHO’s health systems framework defines the system using six building blocks [20] and has been used to assess health systems performance, examine interactions between health reforms and country health systems, and explore implications of health sector reforms [21–23]. Bryce et al. proposed an evaluation framework that examines general socioeconomic and other contextual factors, along with health systems and concurrent programmes that might interact with the programme of interest and affect programme implementation and effectiveness in achieving its desired health outcomes [21, 22]. UNICEF developed its District Health System Strengthening framework to unpack how, at subnational level, service delivery must be managed in order to achieve high effective coverage [23]. Overall, the literature on health systems thinking has grown substantially [24]; there is a continued need to examine both what is needed across levels of a health system as well as the elements within building blocks to ensure that specific interventions reach those in need in a timely manner and with sufficient quality to have health impacts [23, 25, 26]. This kind of systems thinking is essential to identify the changes required to achieve impact.

While increasing emphasis is placed on strengthening health systems and the contexts within which interventions are delivered [27, 28], the mechanisms for how to do so successfully still remain elusive. Health systems strengthening requires an understanding of the underlying supply and demand side mechanisms and the interplay of system components and structures in influencing effective coverage of interventions. Nilsen has proposed a typology of theoretical frameworks and approaches used in implementation science [29], categorising them into those used to guide translation of research into practice, those that clarify what influences implementation outcomes, and those that evaluate implementation. What is missing, however, is a single approach that can be applied across planning, monitoring and evaluation to facilitate implementation and course correction at all levels of the health system.

This paper describes such an approach, termed the Pathway to High Effective Coverage (hereafter referred to as the Pathway), designed to outline and understand a comprehensive set of elements influencing effective coverage. We describe the Pathway’s development and its application in the context of scale-up of newborn health interventions under the Saving Newborn Lives (SNL) programme. We first describe the rationale for and the process used to develop the Pathway; then, we note how we used it to support collaborative strategic planning, monitoring, evaluation and adaptive management. We also describe how we used the Pathway to catalyse efforts of policy-makers, managers and external partners to scale-up evidence-based interventions to achieve maximal impact.

## Methods: rationale and process of development of the Pathway to High Effective Coverage

### Context: Saving Newborn Lives

Save the Children’s SNL programme is a globally recognised leader in newborn health [30, 31]. Since 2000, SNL has raised awareness about the high burden of preventable newborn deaths, generated and provided evidence on how to prevent these deaths, and supported low-income countries to reach the most vulnerable newborns with lifesaving interventions. A 2012–2013 internal evaluation of SNL (unpublished) highlighted the importance of addressing implementation strength of newborn health interventions within existing systems to ensure effective coverage at national scale of populations’ at need. The next phase of SNL (SNL3, 2013–2018) operated in seven countries across two regions, with an aim of learning what it would take to deliver these evidence-based interventions at national levels of high effective coverage within existing public health systems. Along with this aim was a mandate to develop and apply a measurement strategy that captured both implementation strength and effective coverage.

During 2013–2014, SNL and its evaluation partner, EnCompass LLC, applied an iterative approach to create, test and fine-tune the Pathway, seeing it as a ‘living’ framework to be re-examined and refined. This framework built on prior SNL efforts to define and measure national level readiness to scale-up and rollout newborn health interventions [32] and drew on work by others, particularly with regards to defining implementation strength and effective coverage [9, 10, 23, 33]. Altogether, between early-2013 and mid-2014, we undertook multiple reviews of published and grey literature, external consultations, and internal discussions to refine the Pathway. Each version of the Pathway was tested conceptually using specific newborn health interventions in SNL country programmes (more details provided in the Results section).

### Initial literature review and conceptualisation

We formed a core team comprised of SNL and EnCompass staff engaged in work on measuring and understanding effective coverage. EnCompass conducted an initial review of the literature, including reports and papers, to inform the framework’s development. The initial framework included national readiness, implementation strength, effective coverage and impact reflecting SNL’s prior work on national scale-up readiness benchmarks [32], WHO’s health systems building blocks [20], and the implementation strength concepts, including work from the African Health Initiative [33] and others [34].

The Pathway’s development sought to shine light into the ‘black box’ between policy/national programming inputs and effective coverage by unpacking implementation strength. While all SNL3 interventions were related to newborn health, the interventions and packages themselves, and their intended delivery platforms, varied substantially across the contexts of the seven countries in which SNL3 was working. Therefore, the operationalisation of implementation strength had to be ‘unpacked’ and tailored to the particularities of the context and the health systems in which it was to be delivered.

Subsequent internal consultations attained consensus on working definitions of the key concepts of effective coverage and implementation strength, while also revealing conceptual gaps in linkages between national level readiness (policies, guidelines, indicators, competencies) and implementation strength at the point of intervention delivery. As a result, two additional components – system structures and management capacity – were incorporated to illustrate how the original concepts connect with health systems infrastructure and the subnational management structures, particularly as applied to the contexts in the seven countries where SNL was working. Internal consultation also highlighted the need to expand and detail elements within two dimensions of implementation strength tied to (1) whether key programme elements and processes are in place at the point of intervention delivery (process) and (2) the extent to which the platform is functioning and interventions delivered (outputs). SNL and EnCompass explored additional literature [9, 10, 12, 23, 24, 35–40] and sought inputs by the Johns Hopkins Institute for International Programs team engaged in the African Health Initiative to refine these areas of the Pathway. Additional external consultations were held with thought leaders in measurement and maternal, newborn and child health interventions and systems, who underscored the need for such a framework prompted further exploration of what elements needed inclusion and the extent to which these could be sufficiently defined and measured.

## Results: the Pathway and its applications

### Description of the Pathway

**Fig. 1:**
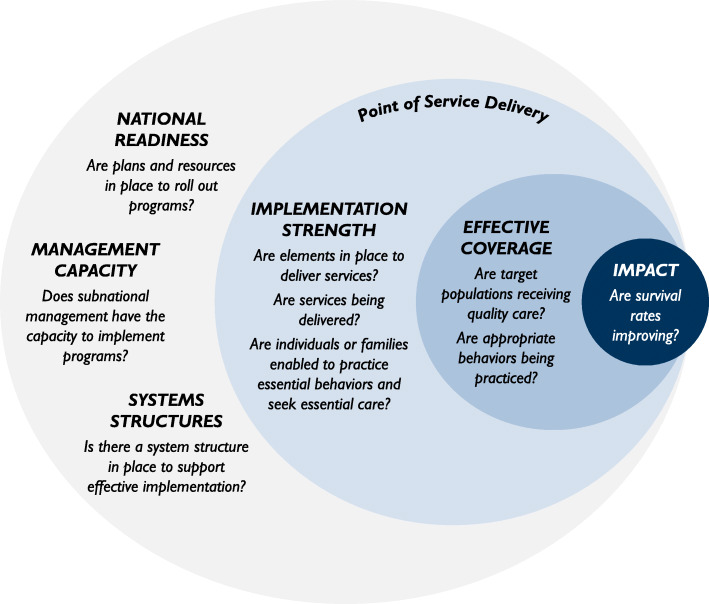
The six components of the Pathway to high effective coverage

The Pathway to High Effective Coverage, as applied starting in 2014, is shown in Fig. 1. It has six components – (1) national readiness; (2) system structures; (3) management capacity; (4) implementation strength; (5) effective coverage; and (6) impact. Note that components 1 and 2 are situated at a national level, while component 3 is at subnational level; component 4 operates at the point of intervention delivery, which can be at facility or community level (e.g. postnatal care), a community mechanism (e.g. household visits conducted by community health workers), or a caregiver (e.g. parental care-seeking practices). Visually (as seen in Fig. 1), impact sits partly but not entirely within this framework, in recognition that factors external to health systems also impact health and survival [19, 41]. Although not shown in Fig. 1, the Pathway itself sits within a larger social, geopolitical and economic context that influences health systems.

### Pathway components

#### National readiness

This component drew heavily from work on the scale-up readiness benchmarks [32]. Its elements explore the extent to which the intervention or intervention package (either community or facility-based or both) has been integrated into national systems and is reflected in policies, plans and resources. National readiness also reflects some of the elements from the WHO building blocks.

#### Systems structures

This component explores the platforms through which the intervention(s) will be delivered and whether they are in place and sufficiently resourced. This component drew heavily from the WHO building blocks framework but is expanded to ensure that it would be relevant for facility and community-based interventions.

#### Management capacity

This component looks at the systems and structures at subnational level (e.g. state, province, region, district) that are required to take on management of intervention delivery. This component is particularly important for national scale-up to be effective and may vary substantially within a country. This component drew heavily from UNICEF’s work on District Health System Strengthening [23].

#### Implementation strength

This component is situated at the point of intervention delivery. We conceptualise implementation strength as the process and outputs components of traditional logic models and monitoring and evaluation frameworks, which are structured around elements of inputs–processes–outputs–outcomes–impact. We aim to measure the extent to which interventions are actually implemented and whether the processes are in place and producing the outputs expected. Definitions and the elements of implementation strength emerged initially from the work of Johns Hopkins University Institute for International Programs and Informed Decisions for Actions in Maternal and Newborn Health [9, 10, 42, 43].

#### Effective coverage

This component expands the notion of coverage from simply a contact to one which captures aspects of quality produced by strongly implemented programmes. The quality of interventions is critical to produce the health outcome of interest. The SNL and EnCompass team expanded the effective coverage component to include not only preventive and curative interventions but also household practices that produce health impacts. This component aligns with the work of Tanahashi [2], Victora et al. [22], Bryce et al. [21], Peters [44], and others [4, 7, 35, 45].

#### Impact

The Pathway assumes that interventions delivered through health systems, both at facility and community points, are contributing to reductions in morbidity and, ultimately, mortality. While not addressed in the Pathway directly, the broader social, political, geographic and cultural contexts in which health systems are situated are recognised as important influences on morbidity and mortality [36, 41].

**Fig. 2:**
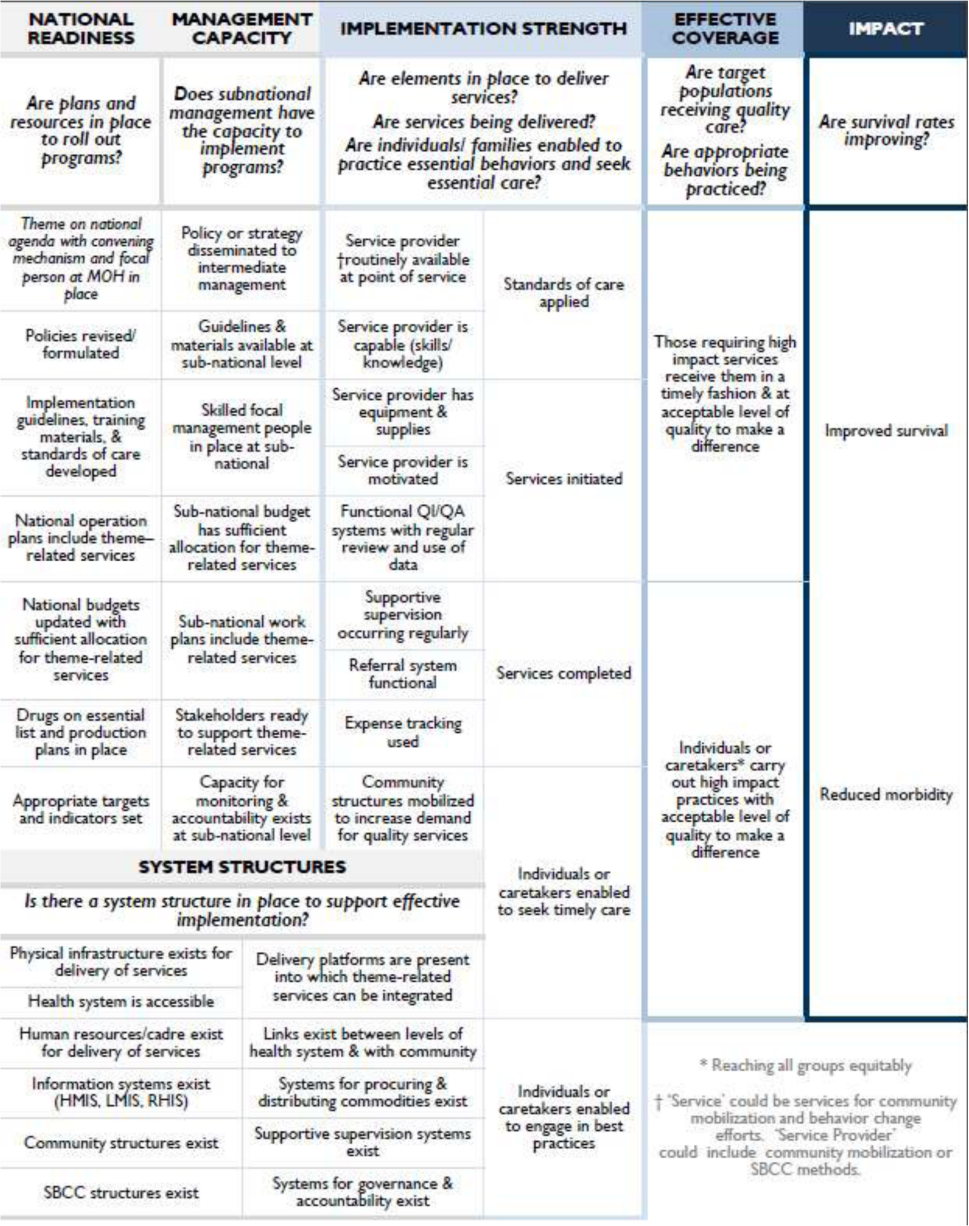
Elements mapped to the Pathway to High Effective Coverage

Forty elements make up the six components of the Pathway, as presented in Fig. 2 each element is further defined in Table 1, which also highlights possible data sources for measuring level of achievement. Ultimately, for any health intervention in any context, we believe that all relevant elements need to be addressed to achieve high national levels of effective coverage. These elements provide points of action and points of operationalisation for strategic planning, monitoring and evaluation across various stakeholders.

**Table 1:** Definitions of elements and potential data sources

Pathway component and related elements	Potential measurement data sources
**NATIONAL READINESS:** This component explores the extent to which the service or service package (either community or facility-based or both) has been integrated into national systems and is reflected in policies, plans and resources; national readiness also reflects some of the elements from the WHO building blocks	
**Thematic area on national agenda with convening mechanism and focal person at Ministry of Health (MOH) in place:** Technical working/advocacy group established and advocating for health thematic area OR existing working/advocacy groups have integrated the health thematic area into their terms of reference; there is also a focal person established or identified, housed within the MOH, and the position is funded	Document review, e.g. national health policies and plans, national budget or national health accounts, routine health information system data, national health surveys, Every Newborn Action Plan scorecard Key informant interviews (global and country level)
**Policies revised/formulated:** National theme-related policies have been updated and/or developed to reflect the latest evidence on key theme-related health services	
**Implementation guidelines, training materials and standards of care developed:** Specific guidelines, training materials and standards of care for theme-related health services have been created and approved	
**National operation plans include theme-related services:** National operational plans for the health sector include specific mention of implementation of key theme-related health services	
**National budgets updated with sufficient allocation for theme-related services:** National budgets have been created for national operational plans that include allocations for implementation of key theme-related health services	
**Drugs on essential list and production plans in place:** National essential drug lists include commodities required for key theme-related health services; in addition, plans exist for the procurement of key commodities, including supply forecasting and, where appropriate, production plans	
**Appropriate targets and indicators set:** National strategies and policies have established effective coverage targets for key theme-related health services. Indicators to track key theme-related health services have been incorporated into national systems (e.g. health management information system, national surveys)	
**SYSTEMS STRUCTURES:** The component explores the platforms through which the service(s) will be delivered are in place and sufficiently resourced	
**Physical infrastructure exists for the delivery of services:** Adequate physical structures, such as buildings or mobile units, exist with sufficient space to accommodate the delivery of the services of interest	Document review: routine health information system, human resource information system, Every Newborn Action Plan scorecard, project reports, evaluations, studies, and household/health facility surveys, guidelines (supervision, community health worker) Key informant interviews (country level)
**Health system is accessible:** The healthcare delivery system is accessible to the population in need of services. Accessibility includes distance/availability of transport, costs and availability of culturally and linguistically appropriate service delivery to the population in need of services	
**Human resources/cadre exist for delivery of services:** Cadres of health providers exist, who can be used for the delivery of services; for example, there exists a cadre of community health workers who could take on home visits for provision of care	
**Information systems exist:** Systems exist to manage information on health service delivery: Routine Health Information System, Logistics Management Information System and Human Resources Information Management System, into which health area-specific information can be incorporated	
**Community structures exist:** Community structures exist that could be leveraged for social and behavioural communication efforts.	
**Social and behavioural change communication structures exist:** Specific structures exist for the provision of social and behavioural change communication such as radio stations or other media stations, local theatre groups, social media	
**Delivery platforms are present into which theme-related health services can be integrated:** A delivery platform organises services around similar work, rather than areas of specialisation; for example, existing delivery platforms, such as antenatal care, labour and delivery, could be important for the provision of services for newborn care; this includes platforms at the community level such as community health workers	
**Links exist between levels of health system and with the community:** Linkages that permit referral and cross-referral exist between different levels of the health system and between the health system and communities	
**Systems for procuring and distributing commodities exist:** Systems for forecasting, procurement and distribution of health-related commodities (e.g. medications and other supplies) are in place, into which required commodities for theme-related care can be incorporated	
**Supportive supervision systems exist:** There is a system to provide supervision that incorporates an interactive relationship between supervisors and supervisees, including mentorship, joint problem-solving and active communication	
**Systems for governance and accountability exist:** A mechanism is in place to hold health systems accountable across levels of the national structure and functioning structures that permit governance of the health system across multiple levels	
**MANAGEMENT CAPACITY:** The component explores the systems and structures at subnational level (e.g. state, district, province), that are required to take on management of service delivery; this component is particularly important for national scale-up to be effective and may vary substantially within a country	
**Policy or strategy disseminated to intermediate management:** National policies and strategies related to the health thematic area have been disseminated from the national level to intermediate, sub-national management levels	Document review: routine health information system, human resource information system, project reports, studies, and household/health facility surveys Key informant interviews (country level)
**Guidelines and materials available at sub-national level:** Guidelines and materials related to the health thematic area, developed at national level, have been disseminated from national to intermediate, sub-national management levels	
**Skilled focal management people in place at sub-national level:** Staff with theme-related health knowledge and skills are in place at sub-national level to serve as focal points for the implementation of national policies, standards and practices	
**Sub-national budget has sufficient allocation for theme-related health services:** Budgets at sub-national level have been developed with sufficient allocations to permit appropriate implementation of theme-related related services, as per sub-national work plans	
**Sub-national work plans include theme-related health services:** Workplans at sub-national level have been developed to reflect national priorities for thematic health area, including key services	
**Stakeholders ready to support theme-related health services:** Key stakeholders at subnational level have been sensitised and mobilised to support the implementation of theme-related health services	
**Capacity for monitoring and accountability exists at sub-national level:** Staff with adequate skills and knowledge are in place to implement national monitoring systems for theme-related health services; accountability mechanisms, including local governance structures and community accountability structures, where relevant, are in place and actively incorporate review of theme-related services	
**STRENGTH OF IMPLEMENTATION:** This component is situated at the point of service delivery; the component corresponds to the process and output components of traditional logic models and monitoring and evaluation frameworks that are structured around inputs–processes–outputs–outcomes–impact; the aim is to measure the extent to which intervention elements are implemented, such as whether the processes in place are producing the outputs expected	
***Elements in place to deliver services:*** At service delivery level, the processes and materials are in place and functioning in order to deliver the service(s).	
**Service provider is routinely available at service delivery point:** Sufficient numbers of defined category of providers (or services, e.g. media outlets) are available at appropriate delivery points (e.g. facilities, health posts, communities)	Document review: routine health information system, project reports, evaluations, studies and health facility surveys Key informant interviews (country level)
**Service provider is capable (skills/knowledge):** Specified cadre(s) of providers or service outlets (in the case of media, for example) have received training (initial/refresher) for the specific service, which includes assessment and hands-on practice, and have the knowledge and skills required to provide high quality services	
**Service provider has equipment and supplies:** Specified cadre(s) of providers have the necessary equipment (including all supplies and medicines) to provide quality services as delineated in programme guidelines; the supplies and equipment need to be routinely within access and functional (not expired in case of medicines) and in adequate quantities for the numbers of providers identified for service provision and for expected services delivered	
**Service provider is motivated:** Specified cadre(s) of providers willing and enabled to provide services that they are trained and equipped to do	
**Functional quality improvement/quality assurance systems with regular review and use of data:** Systems are in place to ensure that the programme can ensure that quality services are provided, including systems for quality improvement and quality assurance, routine monitoring to ensure data are available for accountability, supervision and programmatic decision-making	
**Supportive supervision occurring regularly:** Supportive supervision visits are being received by service providers engaged in the intervention of interest at regular intervals and provide feedback and support specific to the intervention(s) of interest	
**Referral system functional:** Referral processes are in place for referrals between facility levels and between facility-based and community-based programmes; this includes defined processes for where to refer, how to refer, how to document appropriately, and where and how to counter-refer	
**Expense tracking used:** Use of resources at the local level are being tracked to ensure adequate resources for continuation of services	
**Community structures mobilised to increase demand for quality services:** Existing community structures (e.g. women’s groups, civil society, etc.) are actively engaged in improving services (e.g. availability and quality) and enabling caregivers to seek care and use best practices for newborns	
***Programme Functioning:*** This describes what is occurring if services are being delivered; it captures the basic outputs of knowledge, practices and services provided	
**Standards of care applied:** Service delivery follows quality standards	Document review: routine health information system, record reviews and observations to assess quality and adherence to standards of care Project reports, evaluations, studies, household surveys Key informant interviews (country level)
**Services initiated:** The population in need initiating care or treatment for complications or receives preventive services or practices that prevent complications and promote health (this represents the first contact)	
**Services completed:** The target population or population in need initiating care or treatment or preventive services and receiving the full course or ‘dose’ (this includes full set or completed services)	
**Individuals or caretakers (parents or guardians) enabled to seek timely care:** Individuals or caretakers are aware of services available and are enabled (know when/how) to access care from appropriate service providers (this includes knowing what services are available and being able to access)	
**Individuals or caretakers (parents or guardians) enabled to engage in best practices:** Caretakers are aware of and enabled (know how/why) to use best care and treatment practices to treat complications or promote health	
**EFFECTIVE COVERAGE:** This component takes coverage from a contact to capture aspects of quality produced by strongly implemented programmes in order to produce the health outcome of interest as well as appropriate reach to those in need of the intervention; effective coverage captures both preventive and curative services as well as household practices that produce health impacts	
**High impact quality services received:** Target population receives high impact services in a timely fashion and at acceptable level of quality to make a difference	Document review: household surveys, quality of care surveys (observations, medical record reviews, client exit interviews)
**High impact quality services provided:** Individuals or caretakers carry out high impact practices with acceptable level of quality to make a difference	
**IMPACT:** This component assesses changes in morbidity and mortality as a result of the delivery of service(s) and/or changes in behaviours and care-seeking	
**Improved survival:** Reductions in mortality of the target population of the health area that are a result of the delivery of the service(s) and/or changes in behaviour and care-seeking	Document review: household surveys, routine health information systems; special studies (e.g. mortality, verbal autopsy studies)
**Reduced morbidity:** Reductions in morbidity among the target population of the health area that are a result of the delivery of the service(s) and/or changes in behaviour and care-seeking

### Applications of the Pathway

In 2014, SNL began to use the Pathway for strategic planning, monitoring implementation strength and effective coverage, and evaluation for specific newborn health interventions in its focus countries. SNL and EnCompass continued to refine the elements for clarity and specificity when necessary. The following are broad examples of how the Pathway was used in strategic planning, monitoring and evaluation.

#### Use in strategic planning

At the start of SNL3, the programme strategically identified specific newborn health interventions to prioritise within each of its seven countries. In 2014, EnCompass facilitated a review process in each country with the SNL teams and, subsequently, with local stakeholders to assess those priority interventions using the Pathway to identify achievements, gaps and to determine what was required to achieve effective national level scale-up. During this process, SNL country teams first assessed each element for whether it was ‘achieved’, had plans in action to address it, or needed attention. The SNL teams then identified next steps for their own actions and areas where others were better placed to influence progress. This was followed by a similar, collaborative process with local stakeholders, including government representatives, civil society organisations, academic institutions, professional associations, multilateral agencies, and local and international implementation partners.

In Uganda, participating civil society organisations identified and tackled the lack of harmonisation of their efforts, while those in academia began to visualise where within the Pathway their efforts were contributing towards effective scale-up. Both groups further identified their next steps accordingly, which included a research prioritisation exercise informed by the Pathway [37]. In Nigeria, SNL along with national stakeholders mapped progress related to specific elements along the Pathway for chlorhexidine application for umbilical cord care, antenatal corticosteroids for preterm births, and management of possible severe bacterial infections in newborns. This review of progress across all three areas allowed SNL to focus its scale-up efforts on chlorhexidine, based on the progress and focus of other partners on this intervention (e.g. two large USAID-funded projects) and recognising that the health system would require assistance at both the federal and the state levels. In Nepal, the SNL team assessed progress on chlorhexidine use and essential newborn care at both national and district levels, and then convened a meeting with key implementers, senior Ministry of Health representatives, and other partners to present and discuss progress. The Nepal SNL team and partners used this assessment to plot a way forward, ultimately revisiting this plan after the 2015 earthquake to examine how the system was affected and identifying required course corrections. Positive perceptions of the utility of the Pathway emerged from internal review stakeholders.

“*The Pathway analysis is one of the effective tools to evaluate ourselves at national context. This process is good to identify the national need to focus and strengthen the content*.” National stakeholder, academic institution and professional association member, Nepal

“*The Pathway is very good guide for reviewing and planning. In the areas we have proceeded definitely there are more challenges than successes … We really need to see how we can translate those successes under national readiness into strengthening implementation*.” National stakeholder, international partner, Nigeria

#### Use in monitoring implementation strength and effective coverage

SNL used the Pathway to frame the monitoring of implementation strength and progress towards effective coverage in several countries. The measurement focus and approach varied, based on country priorities, SNL3’s strategic priorities and interventions, and areas identified as critical for influencing the achievement of high effective coverage (separate paper forthcoming detailing the approach and outcomes). The Malawi case study (Box 1) provides more details on how plans developed; a global presentation captures our data gathering, analysis and use experience [38]. In Bangladesh, we carried out a similar process to identify inputs necessary for the implementation of a comprehensive newborn care package to be delivered through the public health sector. Unlike in Malawi, in Bangladesh, the SNL team and newborn care stakeholders identified indicators for all elements within implementation strength and attempted to monitor all elements in one district where the package was being piloted.

#### Use in evaluation

The Pathway was used in designing the midterm and final evaluations of SNL3 and two post-hoc evaluations in countries where SNL had previously operated but was no longer active (Box 2). In these instances, the Pathway served to frame and guide data collection and analysis and, in collaboration with stakeholders, inform conclusions and next steps. The evaluation teams triangulated evidence of progress within each Pathway element using a broad range of data sources. These (also shown in Table 1) included, but were not limited to, semi-structured interviews with country-level informants, national and global documents, SNL project documentation and analyses, and national level data on utilisation and coverage (Routine Health Information System, Demographic and Health Survey, Multiple Indicator Cluster Survey and other partner surveys where available). In all cases, evaluation teams convened local stakeholders to present emergent findings by Pathway elements to validate findings, elucidate emerging themes, jointly develop conclusions and discuss the way forward.

Box 2 Use in Evaluation: Application of the Pathway in in Mali EnCompass applied the Pathway in 2016 as the framework for a post-hoc evaluation in Mali (an SNL 2 country) 4 years after SNL had left the country. The Pathway provided the conceptual frame for data collection and analysis. The level of evidence varied across elements; the evaluation team was able to leverage multiple existing data sources, such as several Demographic and Health Surveys and Multiple Indicator Cluster Surveys, studies and evaluations carried out in previous and current newborn health projects, and complemented these sources with 21 interviews with key in-country stakeholders. All data were coded according to the Pathway elements and analysed on a level of achievement (see HEATMAP). During a final workshop, key country level newborn stakeholders reviewed, discussed and validated the findings related to each element and the Pathway overall, and discussed the next steps. The Pathway provided a visual dashboard — a heat map — that clearly highlighted where more needed to be done. As seen in the heat map subsection on national readiness, Mali made important progress between 2000 and 2012 in relation to national preparation to scale-up, which was further consolidated between 2012 and 2016. While many interventions had taken place with the support of partners in specific geographic areas, these are not yet at scale, as reflected in the sections on management capacity and programme elements in place. There was more work to be done at programme functioning to reach effective coverage and impact. As a result of this analysis, key stakeholders were reinvigorated to put in place a plan to execute and advance newborn health in the country. The plan included monthly meetings of technical groups to elaborate a Mali Every Newborn Action Plan, extension of sites for care of small newborns, including KMC at lower level facilities, and the integration of the latest WHO guidelines for newborn care into national policies and procedures. Mali has since finalised its 3-year Every Newborn Action Plan and, as of the end of 2018, stakeholders were still holding meetings to discuss these issues.

Heat Map capturing changes in Pathway Elements, Mali

## Discussion

The Pathway is a robust example of health systems thinking, linking health systems and policy factors with those at the point of intervention delivery, in ways that can generate a useful discourse at all levels on (1) what actions need prioritisation to improve effective coverage, (2) how partners – both implementing and policy/government – can best collaborate; and (3) ways in which success should be defined and measured. It contributes to health systems thinking literature by helping us to consider the underlying characteristics and relationships of system components to better understand their functioning and how they can be leveraged to improve intervention delivery [39].

The experiences of applying the Pathway in various countries and circumstances have generated evidence of its utility for a wide range of stakeholders. The evidence supports broad applicability – in strategic planning to identify key gaps and areas to focus policy and programming efforts; in identifying data needs and monitoring progress of efforts for national and subnational level implementation; and in evaluating progress toward high effective coverage of specific interventions and intervention packages. The Pathway’s contribution is the embedding of implementation strength within the broader context of national readiness, health systems structures and management capacity. By incorporating implementation strength, the Pathway connects national and subnational systems capacity and national readiness directly to intervention implementation [11, 12, 33]. Through the specificity of the elements of implementation strength, the Pathway allows for the association with measures of effective coverage and therefore the identification of direct, actionable findings.

At first encounter, some potential global expert and local stakeholder users felt that the Pathway had too much detail. However, as users were exposed to the data and collaborative methods for processing it, they did not find it overwhelming but, instead, found it enlightening, and the majority noted how it easily highlighted where resources, actions or new actors were needed.

de Savigny and Adam [8] noted the need for conceptual frameworks that outline not just what works in intervention delivery within health systems but also how it works and under what circumstances. The Pathway is a way of organising thinking that can be operationalised as an actionable theory of change. It pulls together factors often associated with the delivery of interventions through health systems and encourages systems thinking to see how they might contribute to achieve the intended health outcomes. It is also easy to complement it with other frameworks, such as the Shiffman framework [40], which EnCompass used in its evaluations to understand more deeply the factors behind the level of progress in national and subnational readiness and implementation attained.

Experiences applying the Pathway have demonstrated that it can be dynamic – highlighting the iterative, non-linear and non-temporal nature of health systems strengthening: a range of actors can implement efforts simultaneously to address more than one of the six components. Progress within the Pathway should be reviewed periodically; its application in post-hoc evaluations showed changes in the status of various elements, including national readiness (because new information and actions emerge in the global health community) and other parts of health systems. The 2016 post-hoc evaluation of SNL in Mali found that, while most national readiness benchmarks had been achieved by 2010 [32], some elements within readiness appeared to regress between 2010 and 2016. Further exploration using the Pathway revealed that the Ministry of Health, with the support of global actors, had introduced new newborn health interventions between 2010 and 2016. The change in readiness was not, in fact, due to backwards movement but instead reflected progress to expand the range of newborn care interventions included. The Pathway’s construction aligns well with the iterative nature of health systems strengthening and makes it well suited for strategic planning, monitoring and evaluation.

Because the Pathway focuses on effective coverage as one of its main endpoints, it should be applied for a specific intervention or set of interventions that focus on a similar target group. As an example, many countries have a newborn package of interventions that include a range of target groups, e.g. all newborns for routine interventions, sick newborns for specific interventions. Interventions that target ‘all newborns’ can be combined and the Pathway applied for that ‘package’, because the measures for effective coverage of the composite interventions have the same denominator – all newborns. However, a newborn package of interventions that targets both healthy and sick newborns would need to be unpacked, because they have multiple endpoints defined for effective coverage, with different denominators defining the different populations in need and the different resources, including commodities, that might be required.

The Pathway was originally developed to aid in thinking about how to achieve high effective coverage at national level; however, it is also useful at subnational level. It can be used for interventions being introduced for national scale-up or to fine tune approaches for existing interventions already in place to achieve full effective coverage. The value of the Pathway is that it allows for flexible relationships between health systems building blocks and the variety of levels of the health system at play.

We elaborated and used the Pathway within the context of newborn health and believe that others could apply it to a broader range of topics within health and beyond. The Pathway has evolved to encompass a range of levels and types of interventions, including but not limited to community-based behaviour change interventions, community and primary health centre interventions, secondary and tertiary hospitals, and the links between them. The Pathway’s flexibility allows for adaptation and tailoring to reflect intervention delivery contexts for health, and potentially other sectors as well.

In the contexts in which we have applied the Pathway, we experienced a range of challenges: availability of up-to-date population or service-based data, particularly for measures of implementation strength, effective coverage and impact; access to documentation of government policies and guidelines; and uniform data collection at the decentralised levels. In addition, some elements pose more difficulty for measurement, such as provider motivation. Routine health information systems data are often weak [46] but can be complemented with other secondary data (e.g. national or subnational surveys) or primary data collection through key informant interviews or special studies. Application of the Pathway can contribute to a culture of data use and the focus on implementation strength has contributed to strengthening routine health information systems by highlighting data gaps.

Similarly, measures of impact, reflected in changes in morbidity and mortality, particularly for newborns, are important but of limited availability [47–49]. Population level surveys are carried out only periodically and are usually not powered to capture subnational changes in neonatal mortality. Existing modelling tools, such as the Lives Saved Tools, while useful in the absence of Civil Registration and Vital Statistics or population surveys, assume effectiveness of interventions based largely on effectiveness and efficacy data, and therefore cannot account for interventions delivered at low quality [50]. When the focus is on intervention scale-up, close attention to the factors leading to high effective coverage and, ultimately, to mortality reductions may serve as better indicators of progress in between larger surveys or until civil registration and vital statistics are improved.

We recognise that the achievement of high effective coverage is conditioned by other factors, including a country’s financial resources, economic infrastructure, private–public sector dynamics, organisational culture and social norms. While these factors are not directly represented in the Pathway, the effects will be seen (indirectly) across elements found in the components of national readiness, health systems structures (behaviour change, supervision systems, accountability systems) and programme elements in place (behaviour change interventions, health worker motivation). Further iterations of the Pathway may incorporate more contextual factors, such as those discussed within the context of quality of care [51, 52], and more directly incorporate the concepts of sustainability, resilience and, ultimately, self-reliance. We did not do an exhaustive literature search across all intervention areas or health systems thinking as we developed the Pathway; however, based on the team’s experience and extensive consultations across multiple sectors, we believe it fits a niche that brings research, implementation and evaluation together. In addition, the field was evolving at the time, as evidenced by the increase in number of publications after early 2014.

## Conclusions

Current discussions on effective coverage at global level further highlight the need to understand the elements contributing to achieving coverage with sufficient quality to make a difference to populations in need. The Pathway promotes systems thinking to better understand effective coverage and, ultimately, health impacts. Its comprehensiveness provides a useful structure for collaborative problem solving among a range of health systems actors and for monitoring and evaluating progress for adaptive management. Applications demonstrate its utility and further study of its use can facilitate refinements and adaptations to strengthen its value across a range of interventions, health areas and sectors.

Box 1 Pathway use in monitoring implementation strength and effective coverage: Malawi Although implementation of Kangaroo Mother Care (KMC) was national policy in Malawi by 2005, when Saving Newborn Lives 2013–2018 (SNL3) started in 2013, data on availability and use of KMC were still limited. To address this situation, SNL convened a meeting with national stakeholders in 2015, during which participants mapped KMC progress to-date along the Pathway. The mapping identified that, while national readiness showed many achievements, there were many challenges and bottlenecks remaining around implementation strength, particularly around identification of eligible babies, intervention initiation and follow-up following facility discharge, which were restricting achievement of effective coverage; these components became the focus of KMC monitoring.

Based on this analysis, SNL and stakeholders defined ‘effective’ coverage for KMC (see below), identified data sources for its measurement, and set targets for national coverage and specifically for the 11 districts where SNL had a greater presence. SNL and stakeholders used the Pathway to help prioritise which specific elements within implementation strength to monitor, identifying those that were most important to track and had data either already available or possibly available through routine systems. This resulted in a consensus on five core indicators for monitoring implementation strength of KMC in Malawi.

 **Effective coverage definition:** Percentage of babies born weighing ≤2000 g initiated on KMC at facilities with inpatient KMC (limited  capacity to capture quality beyond reaching population in-need)

 Data sources:

 **•** Numerator: KMC registers and reporting forms in District Health Information System 2 (DHIS2)

 **•** Denominator: Census projections for number of live births, assuming that 10% would be ≤2000 g (eligible for KMC)

 **Implementation strength indicator definitions (all data sourced from routine health facility data):**

 **1.** **KMC initiation rate:** Number of babies indicated on KMC (inpatient and/or ambulatory) per (1) 100 live births at health facility and (2) 100 low-birth-weight/premature babies identified at health facility

 **2.** **KMC referral completion:** Proportion of babies who were initiated on KMC and referred who completed referral and initiated on facility-based KMC

 **3. Survival to discharge:** Proportion of babies initiated on facility-based KMC who are discharged alive

 **4. Death before discharge:** Proportion of babies initiated on facility-based KMC who died before discharge

 **5. Left against medical advice:** Proportion of babies initiated on facility-based KMC who left against medical advice or absconded

Operationalising the Pathway for monitoring meant ensuring the availability of needed data. SNL therefore next focused on strengthening data availability, quality and use. We took advantage of opportunities to incorporate prioritised data elements into planned assessments, including the 2014 emergency obstetric and newborn care facility assessment surveys [53]. Results from the survey were shared at district planning meetings with factsheets tailored for each district; districts were supported to use the data to inform their budgeting and planning [53]. We also worked with Ministry of Health (MOH) counterparts to improve data from routine information systems, simplifying registers and monthly reporting forms, piloting tools, and incorporating the indicators into DHIS2. Between 2015 and 2018, SNL supported the MOH in analysis of DHIS2 data for all districts to track implementation strength and estimate coverage. Where possible, district level discussions of data informed further course corrections.

To address remaining data gaps identified through the Pathway mapping, we carried out implementation research with local KMC champions and the MOH on follow-up care and outcomes after discharge [54], and on approaches to improve intervention quality through adherence to KMC practices in facilities and households [55]. Results from these were discussed with stakeholders at facility, subnational and national levels to fine-tune intervention delivery and strategic planning.

## Data Availability

The datasets generated and/or analysed during the current study are not publicly available due to their qualitative nature, which are complex and impossible to fully de-identify given the limited number of key stakeholders in some of the countries; however, they could be available from the corresponding author on reasonable request.

## Abbreviations

KMC: Kangaroo Mother Care
SNL: Saving Newborn Lives
UNICEF: United Nations Fund for Children
